# Small, charged proteins in salmon louse (*Lepeophtheirus salmonis*) secretions modulate Atlantic salmon (*Salmo salar*) immune responses and coagulation

**DOI:** 10.1038/s41598-022-11773-w

**Published:** 2022-05-14

**Authors:** Aina-Cathrine Øvergård, Helena M. D. Midtbø, Lars A. Hamre, Michael Dondrup, Gro E. K. Bjerga, Øivind Larsen, Jiwan Kumar Chettri, Kurt Buchmann, Frank Nilsen, Sindre Grotmol

**Affiliations:** 1 Department of Biological Sciences, SLRC-Sea Lice Research Centre, University of Bergen, Pb. 7803, 5020 Bergen, Norway; 2 SLRC, Department of Informatics, University of Bergen, P.O. Box 7803, 5020 Bergen, Norway; 3 NORCE Norwegian Research Centre, Nygårdstangen, Postboks 22, 5838 Bergen, Norway; 4 Department of Veterinary and Animal Sciences, Faculty of Health and Medical Sciences, University of Copenhagen, Stigbøjlen 7, 1870 Frederiksberg C, Denmark

**Keywords:** Genetics, Immunology, Molecular biology, Diseases, Pathogenesis

## Abstract

Little is known about glandular proteins secreted from the skin- and blood-feeding ectoparasite salmon louse (*Lepeophtheirus salmonis*). The labial gland has ducts extending into the oral cavity of the lice, and the present study aimed to identify novel genes expressed by this gland type and to investigate their role in modulation of host parameters at the lice feeding site. Five genes associated with labial gland function were identified and named *Lepeophteirus salmonis* labial gland protein (LsLGP) 1–4 and 1 like (LsLGP1L). All LsLGPs were predicted to be small charged secreted proteins not encoding any known protein domains. Functional studies revealed that LsLGP1 and/or LsLGP1L regulated the expression of other labial gland genes. Immune dampening functions were indicated for LsLGP2 and 3. Whereas LsLGP2 was expressed throughout the parasitic life cycle and found to dampen inflammatory cytokines, LsLGP3 displayed an increased expression in mobile stages and appeared to dampen adaptive immune responses. Expression of *LsLGP4* coincided with moulting to the mobile pre-adult I stage where hematophagous feeding is initiated, and synthetic LsLGP4 decreased the clotting time of Atlantic salmon plasma*.* Results from the present study confirm that the salmon louse secretes immune modulating and anti-coagulative proteins with a potential application in new immune based anti-salmon louse treatments.

## Introduction

Salmon louse (*Lepeophtheirus salmonis*) is a blood-feeding ectoparasitic copepod infecting salmonid fish species of the northern hemisphere. Salmonid aquaculture is based on rearing of high-density populations, and thus in many areas there has been a substantial increase in susceptible salmon louse hosts. The salmon louse population has therefore increased with a consequential negative impact on both farmed and wild salmonids in these areas^1–4^. This negative impact is a result of lice feeding on the host skin and blood, introducing erosions and light ulcers that can disturb the osmotic balance and stress the fish, eventually increasing the susceptibility to other pathogens^5–9^. High parasitic loads can be detrimental to wild post smolts migrating out the fjords^10–12^, hence farmers must keep the lice levels low to reduce infection pressure on wild salmonid populations. With the emergence of a widespread resistance towards anti-lice chemotherapeutants the use of cleaner fish has increased and new methods such as mechanical- and thermal delousing systems, have replaced medical treatments^13,14^. However, ethical aspects on the use of cleaner fish, and increased treatment mortality associated with the new mechanical methods have increased the need for novel control measures such as vaccines, functional feeds, or marker-assisted selection in fish breeding. Detailed knowledge of salmon louse biology and particularly the host-parasite interaction is fundamental to address this.

The life cycle of the salmon louse consists of eight developmental stages separated by moults^15,16^. The initial two nauplii stages are planktonic and lecithotrophic, whereas in the next copepodid stage the louse can detect a nearby swimming host and attach to its epidermis. Here, the louse passes through the parasitic phase of the copepodid stage, two attached chalimus stages and two free living pre-adult stages before it finally moults to the adult stage. During these parasitic stages, the salmon louse modulates the host immune response to avoid clearance, and when blood feeding is initiated at the mobile stages^17^, anti-coagulation factors are expected to be secreted. This is postulated from knowledge of other well-studied host-parasite interactions such as the ixodid tick, that also feeds on host blood for a prolonged time. Tick saliva has been found to inhibit blood clotting as well as inflammation, dendritic cell (DC) maturation and antigen presentation, T-cell proliferation and activation, neutrophil recruitment, antibody function and complement activation^18^. Similarly, there is some evidence indicating that the salmon louse specifically down modulates the salmonid host immune system. A significant suppression of respiratory-burst and phagocytic activity of head kidney macrophages and an increased susceptibility to viral infections have been observed in salmon louse infested Atlantic salmon (*Salmo salar*)^9,19–21^. In contrast to the more resistant pink (*Oncorhynchus gorbuscha*) and coho (*O. kisutch*) salmon, only a mild inflammation without a well-developed hyperplasia is seen at the juvenile lice attachment site in susceptible species such as Atlantic salmon and rainbow trout (*O. mykiss*)^5,7,22–24^. Even where the frontal filament penetrates the epidermis, inflammation is scarce as long as the lice are present^5,25^, indicating that the lice efficiently dampen immune responses in susceptible species.

The salmon louse exocrine glands are hypothesized to be important in the host-parasite interaction through the secretion of modulatory substances, as some gland types have secretory ducts ending at the host-parasite interface^26,27^. The labial gland, in particular, seems to be important, as it first appears at the infective copepodid stage and has secretory ducts ending distally in the oral cavity of the louse near the mandible teeth^26^. Labial gland products are thus likely to be deposited directly onto the lice feeding site. Nevertheless, little is known about the secretory products of the salmon louse labial glands. As parasitism has developed independently multiple times in arthropods^28^, modulatory proteins are not necessarily expected to be homologous even if they target similar host pathways due to convergent evolution. In the present study, we therefore aimed to identify and characterize hitherto unknown proteins expressed by the salmon louse labial gland. RNA sequencing (RNAseq) was carried out on salmon louse tissue samples with a high labial gland content, and five genes encoding proteins without predicted domains were selected and confirmed to be expressed by the labial glands by in situ hybridization. These unknown genes were characterized, and functional studies were conducted to analyse their putative modulatory effects on the Atlantic salmon immune and clotting response by in vivo gene knock down studies and by in vitro studies applying recombinant or synthetic proteins.

## Results

### Identification of labial gland gene candidates and localization of expression

**Figure 1:**
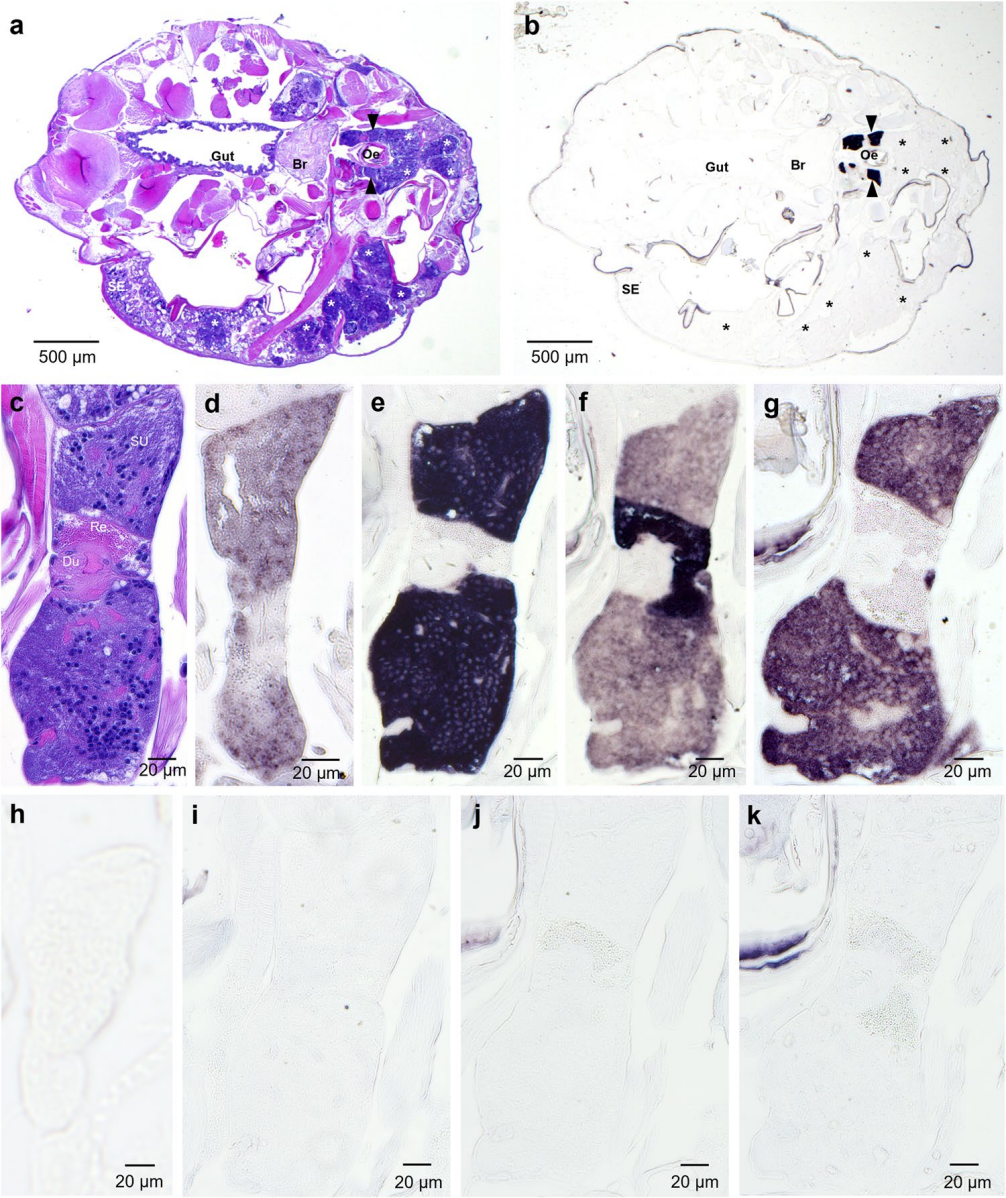
Localization of *L. salmonis labial gland protein* (*LsLGP*) genes by in situ analysis of a pre-adult II female salmon louse. Dark coloring indicates presence of transcripts. (**a**) Overview of the specimen (HE-staining) and (**b**) overview of the specimen hybridized with antisense *LsLGP2* probe, where stars indicate tegmental type 1 glands and arrowheads indicate labial glands. *Br* Primitive brain, *Oe* Oesophagus, *SE* sub-epidermal tissue. A higher magnification of the labial glands treated with (**c**) HE, (**d**) antisense *LsLGP1* probe, (**e**) antisense *LsLGP2* probe, (**f**) antisense *LsLGP3* probe (**g**) antisense *LsLGP4* probe, (**h**) sense *LsLGP1* probe, (**i**) sense *LsLGP2* probe, (**j**) sense *LsLGP3* probe, and (**k**) sense *LsLGP4* probe.

From the RNAseq data, four genes with unknown function were predicted to be expressed in the labial gland of adult lice and thus further evaluated. Transcripts for all four genes, EMLSAT00000009293, EMLSAT00000006642, EMLSAT00000005273 and EMLSAT00000000504 were localized to the labial gland by in situ hybridization, hereafter called *L. salmonis* labial gland protein (LsLGP) 1–4, respectively. Each *L. salmonis* labial gland consists of two morphologically similar secretory units that empty their content into individual reservoirs that further empty through a joint duct^26^. The *LsLGP* transcripts were only localized to the labial gland (Fig. 1), mainly to the secretory unit most distal to the duct (Fig. 1d–g). *LsLGP3* was, however, also expressed by the so-called reservoir unit most proximal to the duct (Fig. 1f). The reservoir unit may thus be more involved in secrete production than first anticipated.

### Characterization of deduced protein sequences

To further characterize the LsLGP genes, RACE was performed for all genes and amplified products were sequenced to ensure that the transcript sequences covered the complete open reading frame (ORF) as predicted from the salmon louse genome.

**Figure 2:**
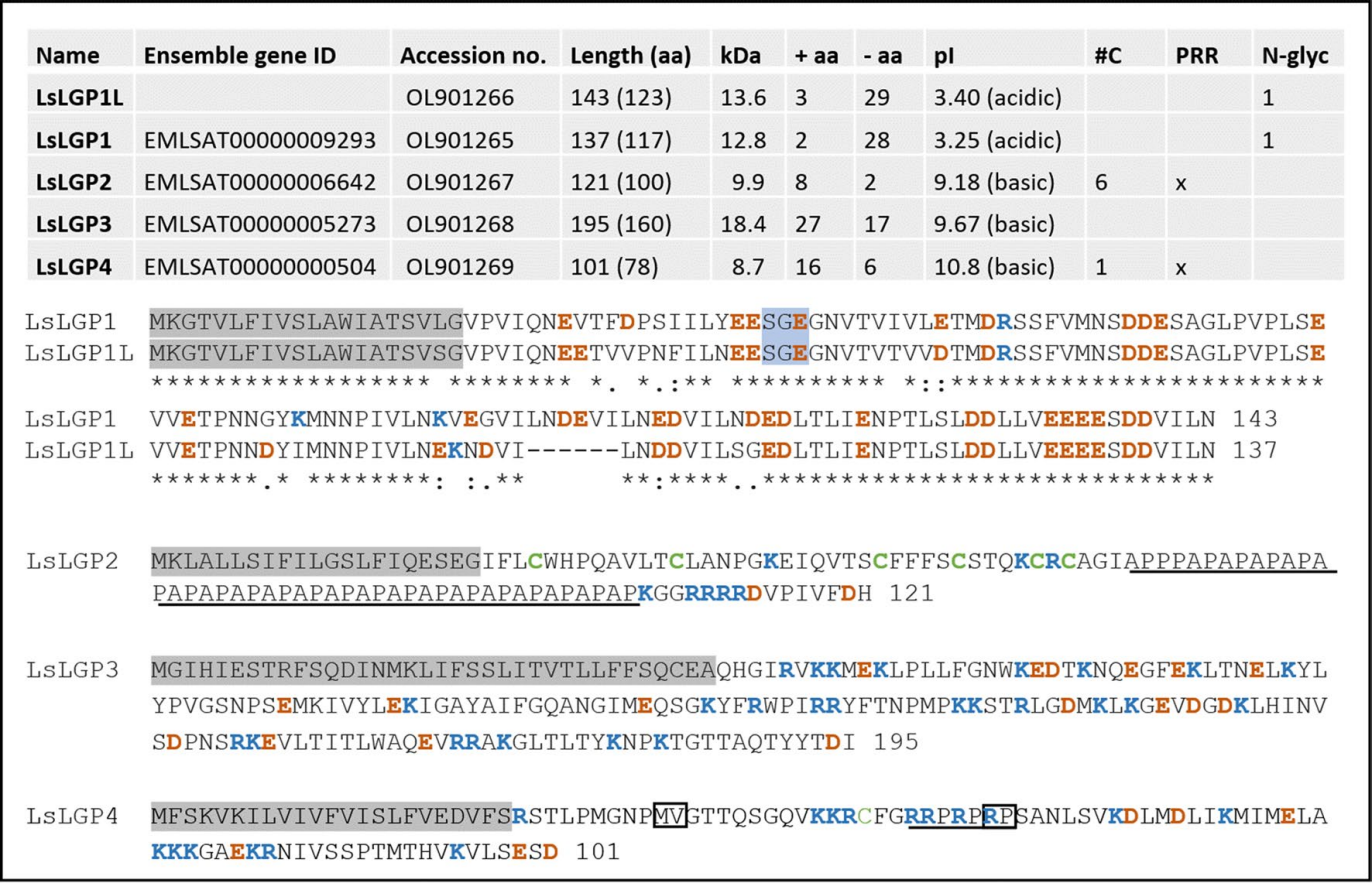
Overview of the predicted *L. salmonis* labial gland protein (LsLGP) 1–4. Upper panel (table): Both the length of the complete ORF and the length of mature sequences (in parentheses) is given, while the putative mass in kDa is given for the mature proteins only. *Aa* amino acids, *pI* theoretical isoelectric point, *#C* number of cysteines, *PRR* proline rich region. Lower panel: Predicted leader sequences are indicated with a grey background, while predicted N-linked glycosylation site are indicated with a blue background. Positively and negatively charged amino acids are shown in blue and red, respectively, while cysteines are depicted in green. PRR’s are underlined, while the two amino acid deletions identified in LsLGP4 is marked with squares. LsLGP1 and 1L are aligned to show identical (*) and similar (: or.) amino acids.

After RACE, all the *LsLGP* genes were found to be short proteins, with sequence lengths predicted between 101 and 195 amino acids (Fig. 2). BLAST searches did not reveal orthologous genes for any of the *LsLGP* genes, and no paralogous genes were identified within the salmon louse genome. Nevertheless, primers designed for *LsLGP1* also amplified a gene highly similar (83%) to *LsLGP1*, named *L. salmonis Labial Gland Protein 1 like* (*LsLGP1L*). *LsLGP1L* was localized to the reverse strand around 21,000 bp up-streams of *LsLGP1* in the salmon louse genome^29^.

Scanning the InterPro database with the putative LsLGP protein sequences revealed no homology to any known protein domains, though all proteins were predicted to contain a leader sequence (Fig. 2). Moreover, all sequences were found to have a high content of charged amino acids (aa’s), where LsLGP1 and LsLGP1L were predicted to be acidic, and LsLGP2–4 predicted to be basic proteins (Fig. 2). In addition to the many negatively charged residues encoded by LsLGP1L and LsLGP1, both proteins were found to contain a high percentage of asparagine and the small hydrophobic residues leucine and valine. Moreover, one N-linked glycosylation site was predicted in both sequences. LsLGP2 was found to contain six cysteines N-terminally possibly forming disulphide bridges, followed by a rather long proline rich region (PRR), APPP(AP)_20_, and a short C-terminus with mainly positively charged amino acids (KGGRRRRDVPIVFDH). LsLGP3 is the largest of the LsLGP proteins, containing no cysteines or other identifiable patterns, though with an overweight of charged amino acids scattered throughout the protein sequence. In LsLGP4, the smallest of the sequenced LsLGPs, a PRR was also found, R(RP)_3_. A total number of 28 clones were sequenced, and of these were four found to have a 6 bp deletion in the PRR region, shortening it to two RP repeats (R(RP)_2_). Another 6 bp deletion was seen in seven other clones in connection to an exon–intron boundary excluding a glycine and an isoleucine. As no paralogue LsLGP4 genes were identified in the salmon louse genome, these transcripts may be considered as allelic variants.

### Ontogenetic expression

**Figure 3:**
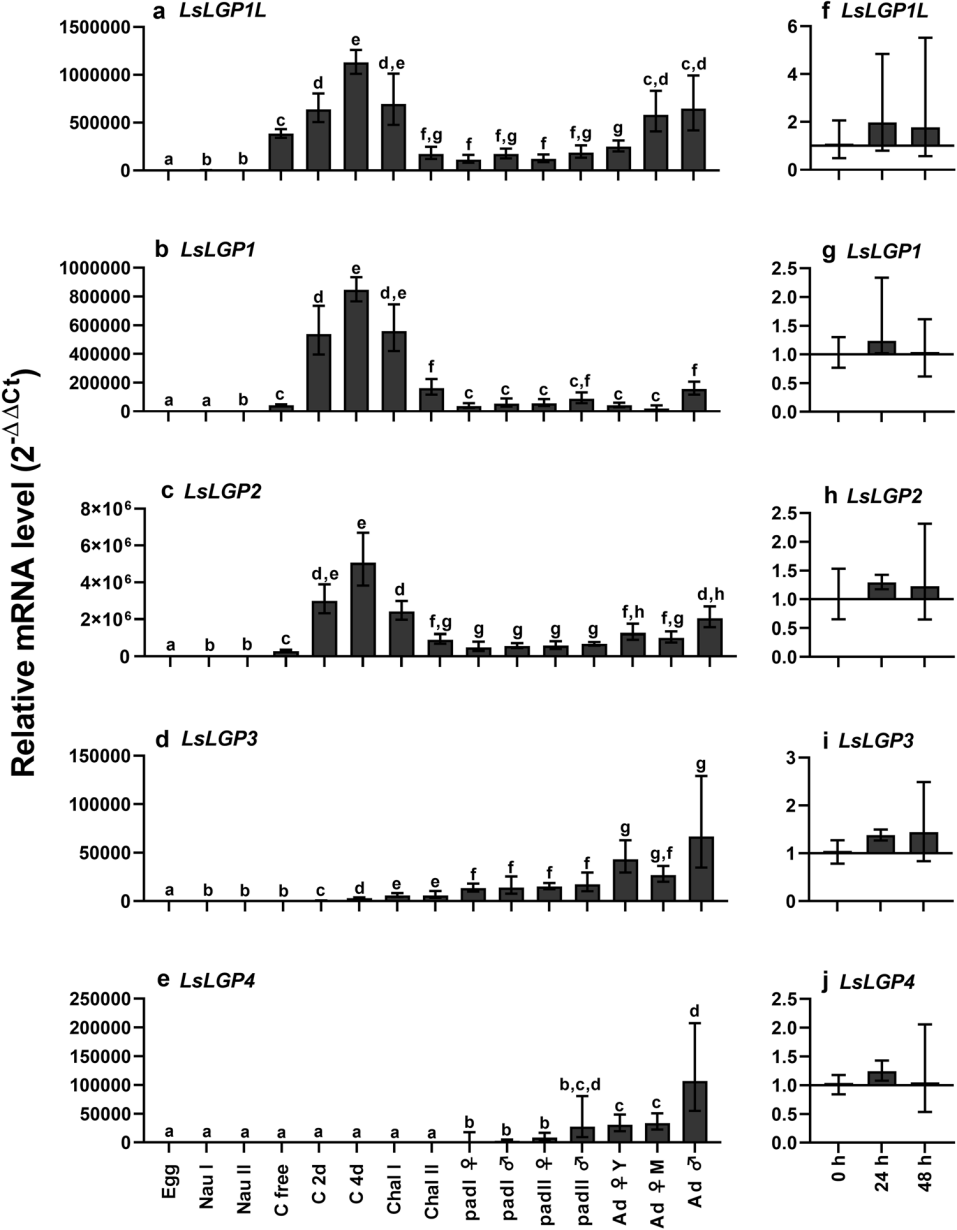
Level of *L. salmonis labial gland protein* (*LsLGP*) transcripts during development and starvation related to the *LsEF1α* and *LsADT3* reference genes (N = 5). (**a–e**) Developmental expression in fertilized egg sacs (egg), nauplius (nau) I and II, planktonic copepodids (c free) and copepodids 2 days (c 2d) and 4 days (c 4d) post infestation, chalimus (chal) I and II, pre-adult (pad) I and II and adult lice (ad). For female adult lice both young (Y) and mature (M) lice were analyzed. mRNA level was related to that of fertilized egg sacs. Statistically significant differences are denoted with different letters (p ≥ 0.05). (**f–j**) Expression in adult females at 0, 24 and 48 h (**h**) after removal from salmon. mRNA levels were related to that of the lice taken directly off the host (0 h).

Generally, the labial gland genes were found to be marginally expressed in eggs and planktonic stages (Fig. 3), though significantly increased transcript levels of *LsLGP1L*, *1*, and *2* were observed in planktonic copepodids (Fig. 3a–c). The levels of these three transcripts further increased rapidly after attachment to the host, with the highest expression detected in 4 dpi copepodids when compared to all other analysed timepoints. During the chalimus II and pre-adult stages, the levels of *LsLGP1L*, *1*, and *2* were significantly lower than during the initial establishing phase of the copepodid, but still significantly higher than in the planktonic nauplius stages. A significant increase was again detected in adult lice, either for both females and males (*LsLGP1L* and *2*) or males only (*LsLGP1*). A steady increase of *LsLGP3* was found during the parasitic stages, with the highest mRNA level detected in adult lice, both females and males (Fig. 3d). *LsLGP4* was found to be the last transcript to increase its expression above that of planktonic stages, with a significant increase detected first when the lice had moulted into the mobile pre-adult I stage (Fig. 3e). The *LsLGP4* transcript level was also found to be at its highest in adult lice, with the highest expression detected in males, likely to reflect the higher labial gland to body size ratio in males. In fact, for all *LsLGPs* besides *LsLGP1L*, a higher average transcript level was observed in adult males compared to adult females.

### Expression in starved lice

As a rapid down regulation of some intestinal transcripts involved in blood-feeding has been detected in starved adult lice^30,31^, we analyzed the expression of the labial gland genes during starvation. The analysis was performed to explore whether the gene products may be involved in digestive processes rather than, or in addition to, host modulation. None of the genes were, however, found to be modulated when lice were incubated off the host for 48 h (Fig. 3f–j).

### Functional studies

#### LsLGP1/1L KD copepodids showed decreased levels of all known labial gland genes

**Figure 4:**
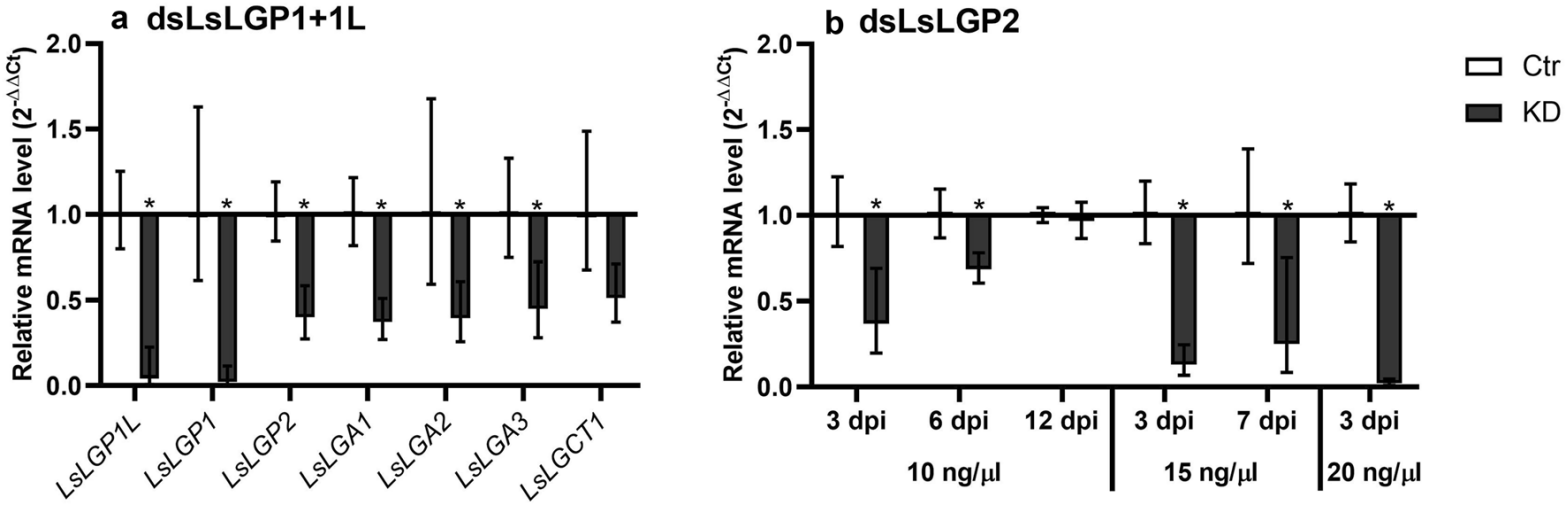
Expression of *L. salmonis labial gland protein* (*LsLGP*) genes after dsRNA treatment. (**a**) Expression of *LsLGP* genes in dsLsLGP1/1L treated copepodids 3 days post infestation related to the *LsEF1α* and *LsADT3* reference genes (N = 8). In addition to KD of the targeted genes, KD of *LsLGP2* and four additional, yet unpublished, genes confirmed to be expressed in the lice labial gland called *L. salmonis* labial gland astacin (LsLGA) 2–4 and *L. salmonis* labial gland chymotrypsin 1 (LsLGCT1), were analyzed. (**b**) Expression of *LsLGP2* in dsLGP2 treated copepodids 3 days post infestation related to the *LsEF1α* and *LsADT3* reference genes (N = 8). Statistical significance was teste with Student’s t-test (p ≥ 0.05) between control and dsRNA treated group and is denoted with an asterisk.

To investigate whether the LsLGP proteins manage to dampen the Atlantic salmon immune response in vivo, double stranded RNA (dsRNA) induced KD of *LsLGP* transcripts was performed in lice followed by subsequent in vivo challenge studies. *LsLGP1* and *LsLGP1L* are highly similar genes, hence, dsLGP1 targeted both genes simultaneously. A KD efficiency of 97.7 and 95.7 were detected for *LsLGP1* and *LsLGP1L*, respectively (Fig. 4a). Interestingly, a significant decreased transcript level of *LsLGP2* was also detected in LsLGP1/1L KD lice, with an efficiency of 60%. We therefore analyzed the expression of four additional genes confirmed to be expressed in the lice labial gland (unpublished data), to further explore the regulative effect of LsLGP1/1L. These transcripts were also found to be significantly decreased in KD animals with KD efficiencies between 49 and 62% (Fig. 4a). Therefore, it was not possible to analyze the specific modulatory role of LsLGP1 and 1L by this approach, as any differences detected could be attributed to any of the other labial gland genes.

#### Infestations with LsLGP2 KD lice induced an increase in immune gene transcripts

**Figure 5:**
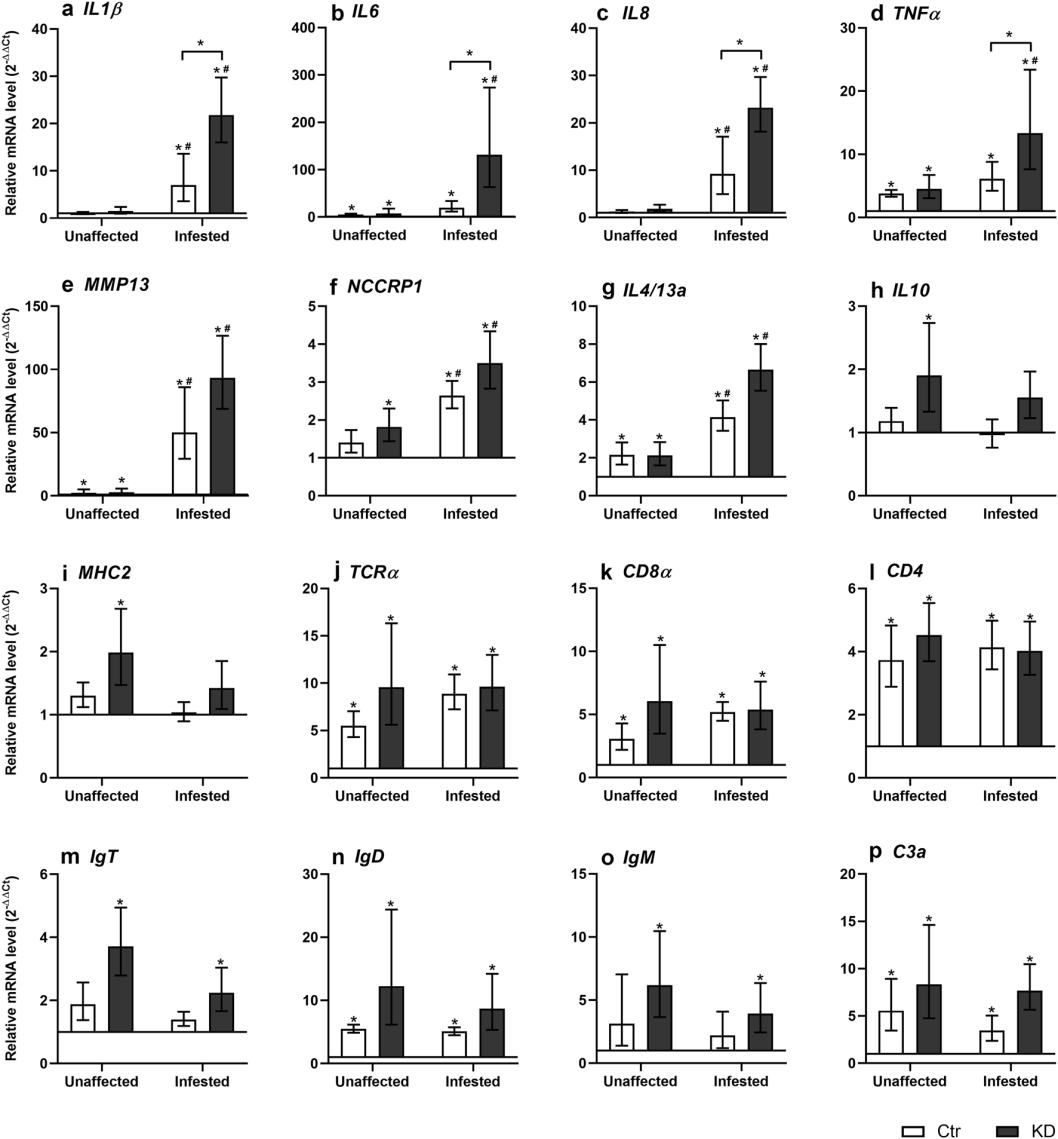
Relative transcript level (2^−∆∆Ct^ ± SD) of selected Atlantic salmon immune gene transcripts in response to control (Ctr) and *LsLGP2* knock-down (KD) copepodids 3 days post infestation. The expression in unaffected and affected sites on infested fish (N = 8) were related to *elongation factor 1 alpha* (*elf1α*) and *tripartite motif* (*trim*) genes (∆Ct) and to non-infested control fish (∆∆Ct). *Indicates significant differences (p ≥ 0.05) when compared to skin from non-infested fish. ^#^Indicates significant differences (p ≥ 0.05) between unaffected and affected skin of infested fish. Significant difference (p ≥ 0.05) between skin sites infested with ctr and KD lice are also denoted with an asterisk (*).

*LsLGP2* was the only labial gland transcript shown to be downmodulated by dsLGP2. The first attempt for *LsLGP2* KD applying normal concentration of dsLGP2^32^ did, however, only result in a transient suboptimal knock down (Fig. 4b). This was also seen when the concentration of dsLGP2 was slightly increased (15 ng/μl), and a doubling of the concentration was required to obtain KD efficiencies above 90%. But, as KD was transient, the immune response against KD lice were only evaluated early when the *LsLGP2* expression was at its highest and KD still sufficient. Expression of immune genes, selected to indicate the involvement of LsLGP2 in inflammation, complement activation and activation of innate and adaptive immune cells, was analyzed in *LsLGP2* KD and control lice infested fish. Immune gene transcripts were analyzed from skin both directly underneath lice (infested site) and in nearby unaffected skin sites at three days post infestation with copepodids. Generally, when comparing immune gene expression in fish infested with control lice to that infested with KD lice, a higher average expression in KD infested fish were seen for all genes analyzed (Fig. 5). The mRNA level of *IL1β*, *IL6*, *IL8*, *MMP13, NCCRP1,* and *IL4/13a* were significantly higher in skin directly underneath the lice in both control and KD infested fish, while the *TNFα* mRNA level were only significantly elevated to that of unaffected sites in KD infested fish. Moreover, a significant higher (p ≥ 0.5) expression was detected for the key pro-inflammatory cytokines *IL1β*, *IL6*, and *IL8*, the anti-inflammatory cytokine *IL10*, and the T helper 2 related cytokine *IL4/13a* in KD infested fish when compared to skin underneath control lice at the site of infestation. While the T-cell response did not seem to be significantly affected by the *LsLGP2* KD, the opposite was indicated for B-cell responses. All immunoglobulin transcript levels were elevated at both unaffected and infested sites in KD infested fish, with a significant increase in KD infested fish seen at both unaffected an infested skin for *IgT* and at unaffected skin only for *IgD*. At the time of sampling, no lice had as expected molted into the chalimus I stage, and similar lice numbers were seen between the control (9.2 copepodids/fish ± 3.9) and KD infested groups (10.8 copepodids/fish ± 2.8).

#### Recombinant LsLGP3 decreased the expression of B- and T-cell markers in primary head kidney leucocytes

As *LsLGP3* and *4* were only expressed in mobile lice stages, adult lice were used for KD studies of these two genes. No differences in the immune response between skin sites infested with KD and control animals were detected in in vivo experiments. This may be due to the mobility of the lice at this lice stage and thus difficulties obtaining samples where mobile lice have been feeding for a prolonged period. Consequently, as an alternative, recombinant LsLGP3 (recLGP3) and synthetic LsLGP4 (synLGP4) was administered to primary head kidney leucocytes in vitro to analyze their immune modulative potential.

**Figure 6:**
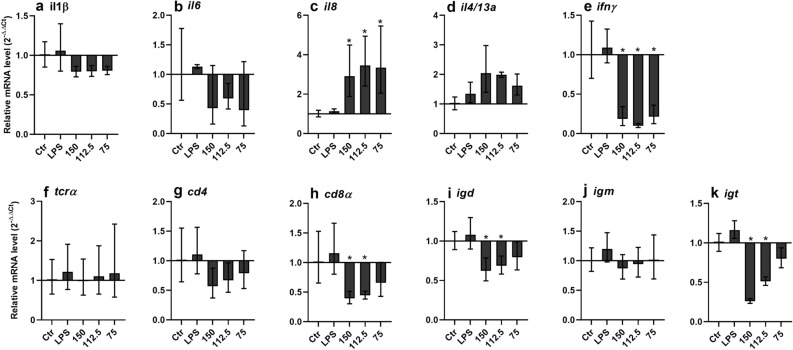
Relative transcript level (2^−∆∆Ct^ ± SD) of selected Atlantic salmon immune gene transcripts in head kidney leucocytes after a 4-h stimulation with recombinant LsLGP3 (recLGP3) (N = 3). Three quantities of recLGP3 were applied, 150, 112.5 and 75 μg/well. The immune gene expression in control (ctr), LPS, and recLGP3 treated leucocytes were related to *elongation factor 1 alpha* (*elf1α*) (∆Ct), using the expression in control cells as calibrator (∆∆Ct). Significant difference (p ≥ 0.05) between ctr and recLGP3 treated leucocytes are denoted with an asterisk (*).

As around 0.3 μg/ml LPS was co-purified together with recLGP3, LPS was also included in the assay both as control and in combination with recLGP3. Analysis of selected Atlantic salmon immune genes in recLGP3 stimulated leucocytes revealed a significant up-regulation of IL8 and IL4/13a, and down modulation of *IFNγ*, *CD8α*, *IgD* and *IgT* (Fig. 6). This down-modulation was not seen in response to LPS alone. No regulation was detected in synLGP4 stimulated leucocytes (Supplementary Fig. S1).

#### Synthetic LsLGP4 inhibit blood coagulation

**Figure 7:**
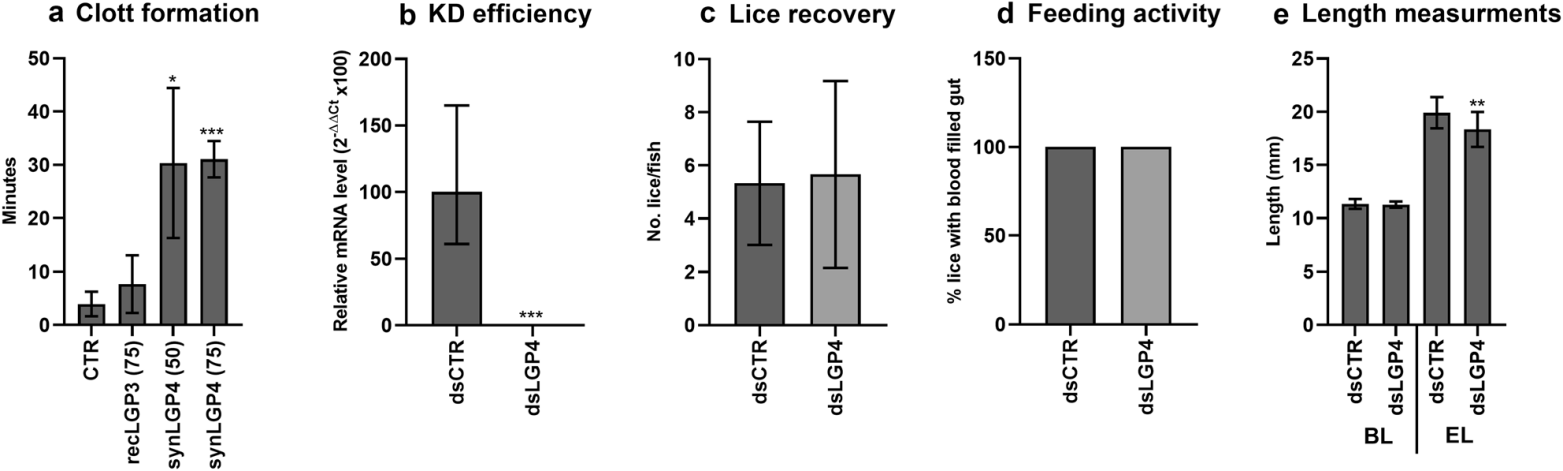
Analysis of LsLGP3 and 4’s role in blood coagulation. (**a**) Coagulation efficiency in control (CTR), recLGP3 (75 μg) and synLGP4 (50 and 75 μg) treated plasma measured as time to plasma gel formation after thromboplastin addition (minutes ± SD; N = 3). Some synLGP4 treated samples did not coagulate during the experiment and was set to 45 min. (**b–e**) Knock down (KD) study of *LsLGP4* during the mobile salmon louse stages to analyse the importance of LsLGP4 in blood feeding. (**b**) Relative transcript level (2^−∆∆Ct^ ± SD) of LsLGP4 in control (dsCTR) and dsLGP4 treated lice at 32 dpi, related to the *LsEF1α* and *LsADT3* reference genes (N = 5). (**c**) Average number of recovered lice per fish (± SD) in fish (N = 3) infested with 10 control or dsLGP4 treated lice/fish. (**d**) Percentage of lice with visible blood in intestine at sampling in control and dsLGP4 treated lice (dsCTR N = 16, dsLGP4 N = 18). (**e**) The body length (BL) and egg string length (EL) of control and dsLGP4 treated lice (mm ± SD, dsCTR N = 16, dsLGP4 N = 18). Significant difference between control and treatment group are denoted with one asterisk (*) if p ≥ 0.05, two asterisk if p ≥ 0.01 and three asterisk if p ≥ 0.001.

As both LsLGP3 and 4 were found to be highly expressed in the mobile stages, their possible inhibition of blood clotting and hence importance in blood feeding were analysed. The prothrombin time test was used to measure blood coagulation, where the clotting time was determined by plasma gel formation in recLGP3/synLGP4 treated plasma compared to the control. SynLGP4 treated plasma demonstrated a significant increase in clotting time compared to the relatively short clotting time in control and recLGP3 treated plasma (Fig. 7a). We therefore conducted a KD experiment injecting pre-adult II female lice and allowing them to develop into adult egg producing lice on fish, to analyse if a reduced LsLGP4 level would affect blood feeding or fecundity at 32 dpi (Fig. 7b–e). An LsLGP4 knock down of 98% did, however, not alter the number of animals with blood in gut. Knock down did also not seem to inhibit anti-coagulation of blood within the gut, as peristaltic movement with liquid blood could be seen to the same degree as in control lice. Moreover, KD did not affect the size of the lice body, though, a small but still significant decrease in the egg string length were detected in the dsLGP4 treated lice.

## Discussion

Blood feeding parasites are expected to secrete factors with immune modulative and anti-coagulative functions, but the presence of such factors have not been confirmed in glandular secretions of the salmon louse. The present study report genes expressed by the labial gland, a salmon louse gland type that is likely to deposit its content directly onto the lice feeding site. These *LsLGP* genes were not found to encode any known protein domains that could help elucidate their function. Signal peptides were, however, identified, indicating that the LsLGPs are secreted. All five labial gland proteins were found to be relatively short, with several charged residues that may act in ligand binding.

The highly similar LsLGP1 and LsLGP1L were both predicted to be acidic proteins at physiological conditions, with a potential role in binding positively charged ligands. As *LsLGP1L* expression was elevated already in planktonic copepodids, it is likely that LsLPG1L is necessary shortly after the copepodids settles on a suitable host. At this stage, the salmon lice attachment and feeding activity inflict tissue damage to the host skin with a low induction of inflammation in susceptible salmonids^5–7,22,25^. In higher vertebrates, extracellular calcium represents one of the earliest signals after tissue damage, functioning as an immune cell attractant and supporting the wound healing response^33,34^. To interfere with this part of the host response, ticks secrete a calreticulin-like protein that also has regions dominated by acidic residues representing putative calcium-binding regions^35^. As Ca^2+^ tend to be bound by oxygen atoms^36^, it is tempting to speculate that the many negatively charged oxygen atoms within the multiple Asp and Glu side chains of LsLGP1/1L may bind calcium ions. Extracellular calcium is also important for keratinocyte differentiation and proliferation, where a low concentration induces proliferation and higher concentrations induces differentiation in higher vertebrates^37^. To regenerate the epidermis, wound healing includes both these processes, where calcium seems to be necessary for normal regenerative skin responses^38^. In the resistant coho salmon, salmon louse induces epidermal hyperplasia that buries and kills the lice^23^, while in the susceptible rainbow trout a milder hyperplastic response is induced underneath mobile lice^7^. Thus, it may be vital for the salmon louse to control the concentration of extracellular calcium to regulate epidermal epithelial cell proliferation. Wound healing is in general a conserved process^39,40^, but little is known about piscine calcium-sensing receptors, and how extracellular calcium affects the fish skin during wound healing and inflammatory responses. Further studies confirming the ligand of LsLGP1/1L is needed, and additional positively charged molecules should also be evaluated. As the KD studies indicated that LsLGP1 and/or 1L are likely key regulators for labial gland secretion, further structural and functional characterization of LsLGP1/1L might provide interesting targets for lice control. Though, if LsLGP1/1L is glycosylated as predicted, eucaryotic expression systems would be necessary to produce functional proteins.

LsLGP2-4 were, in contrast to LsLGP1 and 1L, predicted to be basic proteins at physiological conditions. Additionally, the putative protein sequence of LsLGP2 contains a rather long PRR. As the cyclic five-membered ring of proline is constraining the rotation of its backbone^41^, this long PRR may cause LsLGP2 to be a conformationally loose protein. Proline is a good hydrogen-bond acceptor due to a more accessible and electron-rich carbonyl group than those of other natural amino acids^42,43^. Therefore, (XP)_n_ PRRs are believed to function as rigid and sticky arms that can bind rapidly and reversibly to other proteins^41^, and thus the long PRR of LsLGP2 may work together with the positively charged C-terminus in ligand binding. LsLGP2 KD lice, presumably secreting a lower concentration of LsLGP2, induced a significant increase of the pro-inflammatory salmon transcripts *IL1β*, *IL6*, *IL8* and TNFα compared to control lice. This indicates that LsLGP2 inhibits the action of an immune component or cell directly, or indirectly by binding something that induces inflammation. Moreover, LsLGP2 KD lice induced a significantly higher local expression level of *IL4/13a*, and an increased average expression of *IgM, IgT* and *IgD* was seen at both unaffected and affected skin mainly underneath KD lice. In zebrafish, recombinant IL4/13A promotes Th2-type immune responses by increasing B-cell proliferation and antibody production^44^, while recombinant rainbow trout IL4/13A augments B-cell IgM secretion^45^. It is, however, not known how teleost IL4/13A influences *IgT* and *IgD* expression, although zebrafish IL4 increases the number of IgZ-2 positive (IgZ-2^+^) B-cells^46^. While these cells are IgM^+^IgZ-2^+^ cells, rainbow trout IgT^+^ cells do not express IgM or IgD on their surface^46,47^. Moreover, rainbow trout have three B-cell types expressing IgM and/or IgD; one double positive line expressing both IgM and IgD and two single positive IgM^+^ or IgD^+^ B-cell types^48,49^. IgD single positive B cells have, as well as IgT^+^ B-cells, been suggested to be important in mucosal immunity, and IgT has been identified as a limiting factor for protozoan parasites infecting mucosal surfaces^47–50^. Further study is needed to resolve if the reported increase is of biological relevance caused by an increased expression level of resident B-cells or increased influx of IgT^+^ and IgD^+^ B-cells.

LsLGP3 also seems to modulate the expression of *IgD* and *IgT*, as recLGP3 induced a down modulation of these two transcripts in addition to *CD8α* and *IFNγ* in head kidney leucocytes. A significant reduction of adaptive immune responses has also been indicated in vivo when fish are infested with mobile lice*.* A down modulation of adaptive immune transcripts in both rainbow trout and Atlantic salmon infested with pre-adult and/or adult lice, and an increased susceptibility to infectious salmon anaemia virus in Atlantic salmon infested with mobile lice have been observed^7,9,51^. Interestingly, the expression of *LsLGP3* reaches its highest level in pre-adult and particularly the adult stages. At the adult stage, the lice increase in size, particularly the females that are typically 10–12 mm compared to the 6–7 mm males. Even though adult lice are mobile, the females often gather in groups where they reside at specific locations on the fish typically behind the adipose and anal fins^52,53^. Moreover, the feeding activity of the lice intensify, and it is now breaking through the basement membrane to reach the dermal blood supply inducing an increase in the influx of immune cells to the site of infestation^6,7^. This relatively more intense feeding activity may increase the need to dampen host responses, and the present study indicate that salmon louse labial gland proteins have the ability for such immune suppression. However, LPS contamination represents a possible source of error when studying the immune response towards bacterial derived recombinant proteins, as LPS is a potent immune stimulator^54^. And as a low concentration of LPS was detected in the recLGP3 solution, this could potentially have affected the results in the present study. LPS removal from recombinant proteins requires a rather harsh treatment of the protein with a high potential of altering its function. Therefore, instead of removing LPS from the recLGP3 solution, LPS was included as a control when stimulating the leucocytes, where similar concentration of LPS as what was detected in the recLGP3 solution did not induce any significant regulation of the selected immune genes. This indicates that the co-purified LPS did not induce the down streams effect seen in the recLGP3 treated leucocytes, nevertheless, one should keep this possible source of error in mind when concluding on the present results.

Besides immune responses, coagulation is another important host pathway to suppress for blood feeding parasites. As *LsLGP4* is not expressed before the lice reaches the stage where blood-feeding commonly is initiated, an anti-coagulant role for LsLGP4 could be suggested. This was further supported in an in vitro coagulation test, where a highly significant delay of coagulation was observed in response to synLGP4. The LsLGP4 knock down studies suggested that this protein did not affect the viscosity of the blood within the lice gut, nor the development and growth of the lice. This could indicate that functional amounts of LsLGP4 was not ingested together with the blood during feeding. However, additional gland- or gut derived anti-coagulants are also likely to be secreted by the lice as seen in other blood-feeding parasites such as ticks^18^, and could have masked in vivo effects of LsLGP4 KD. The secretory ducts of the labial glands are found distally in the mouth tube near the mandible teeth^26^, and it is thus likely that the labial gland secrete has its main effect directly at the lice feeding site rather than within the lice gut. This is further supported by the constitutive expression level of the LsLGPs observed in starved lice, in contrast to the decrease typically seen for genes involved in digestion^30,31^. The minor yet significant reduction in egg string length of dsLGP4-treated animals implies, however, that even though LsLGP4 is not essential for a normal feeding activity, it affects the nutritional status of the lice to some degree. Potentially, LsLGP4 KD could complicate the initiation of a blood-meal intake if LsLGP4 is involved in decreasing blood-clot formation at the site of feeding.

Previous studies of salmon louse secretions have focused on secretory/excretory products (SEPs) extracted both from dopamine treated and untreated salmon louse^55–57^. Many components such as astacins, serine proteases, protease inhibitors, calreticulin, and prostaglandin E_2_ (PGE_2_) have been identified in these products, and functional studies have revealed an immune dampening and anti-chemotactic effect of SEPs^56,58^. However, many of the proteins identified in SEPs are proteins expressed by tegumental glands secreting the mucus covering the lice, intracellular proteins or proteins involved in nutritional uptake or digestion, while the LsLGPs identified in the present study could not be identified in the SEP data sets^26,29,55–57^. Probably, the LsLGP’s concentration is low compared to gut and tegumental gland derived proteins, or the release of labial gland secretion is either not constant or not regulated by dopamine but requires host factors. Therefore, this indicate that using salmon louse SEPs in in vitro studies of immune modulation is likely to introduce experimental errors, as digestion enzymes have a low specificity and will non-specifically degrade host immune cells and immune active factors. It is also unlikely that such enzymes are deposited on the host skin during infestation given the low severity of lice induced lesions^6,7,23,25^. Furthermore, it has not been possible to experimentally confirm that lice derived PGE_2_ is in fact taking part in the host-parasite interaction^59,60^, emphasizing that more studies like this, directly addressing the regulation and mode of action of glandular proteins, are needed to analyse the interaction taking place at the site of infestation. New knowledge on this topic may represent a promising fundament for the development of new immune based anti-salmon louse treatments. The present study has identified salmon louse immune modulatory and anti-coagulative proteins and represents an important initial step towards such developments.

## Materials and methods

### Ethical statement

All experiments were carried out in strict accordance with relevant guidelines and regulations. The study was carried out with approval granted from the Ethic committee of *Norwegian Food Safety Authority* (approval numbers 8589) following ARRIVE guidelines.

### Source of *L. salmonis*

A laboratory strain of *L. salmonis salmonis* (LsGulen) was maintained on farmed Atlantic salmon according to Hamre et al.^61^. Salmon were hand fed on a commercial diet (Skretting Nutra Olympic 4.0 mm) and reared in sea water with a salinity of 34.5 ppt and a temperature of 9 ± 0.5 °C if not otherwise mentioned. Eggs, nauplii, copepodids and adult lice maintained off the host were kept in incubators with continuous flow-through of seawater from the same supply as the fish tanks^61^.

All salmon louse stages were collected for ontogenetic analysis in pentadruplicate samples (biological replicates). For the earliest life stages, multiple animals were pooled to yield sufficient total RNA for further analysis as follows: Eggs: 1 eggsac (string) containing approximately 200 developing embryos. Nauplius I, nauplius II and copepodids (free-living): approximately 100 larvae. Copepodids 2 days post infestation (dpi) and 4 dpi: 60 larvae. Chalimus I: 30 animals, chalimus II: 20 animals, female and male pre-adult I and II, and adult stages: single animals. Additionally, both young and mature adult females were collected. Young females were sampled prior to enlargement of the genital segment and the extrusion of the first egg string, while mature females were sampled when bearing external egg strings.

To analyse the expression in starved lice, 15 adult female lice were collected from Atlantic salmon kept at 10 °C. Five lice were analysed from each time point (biological replicates), sampled directly from the host, or after being kept for 24- and 48-h off-host in running seawater.

### Total RNA purification and cDNA synthesis

All tissue/lice samples for RNA isolation were collected in RNA later (LifeTechnologies), kept overnight at 4 ^○^C and further stored at − 20 ^○^C. Total RNA was isolated with a combined Tri reagent (Sigma Aldrich) and RNeasy (Qiagen) method, as previously described^62^, with DNase treatment performed on column. Total RNA from adult female lice were purified with Tri reagent combined with the RNeasy mini kit (Qiagen), while all other samples were purified with Tri reagent in combination with the RNeasy micro kit (Qiagen). Extracted RNA was either kept at − 80 °C until use, or cDNA synthesis was performed directly.

For PCR, cDNA synthesis was carried out using the qScript cDNA SuperMix (Quanta Bioscience), applying 1 µg lice total RNA. For real time RT-PCR, the AffinityScript qPCR cDNA Synthesis Kit (Stratagene) was used according to supplier’s recommendations in a 10 µl reaction, using 200 ng lice total RNA, 1000 ng salmon skin total RNA or 475 ng salmon leucocyte total RNA. Samples were diluted 10 or 5 times for lice and salmon samples, respectively, before storage at − 20 °C.

### RNA sequencing

The region holding the labial gland was dissected out from 20 adult female lice after an over night incubation at 4 °C in RNA later (LifeTechnologies). Total RNA was purified as described in “Source of *L. salmonis*” section. Further library preparation, RNAseq at the Norwegian sequencing Center (Oslo) and data processing were performed as previously described by Eichner et al.^63^. To identify potential labial gland genes, the labial gland expression was compared to RNAseq data obtained for the different developmental stages of the lice, in addition to the gut, gonad and thoracic feet 1 and 2 expression^29^. If expression was high in the labial gland sample, and low or not detected in planktonic stages as well as gonads and gut, the expression were further compared to the expression in the thoracic feet where all types of tegumental glands are found^26^. As tegumental type 1 and 2 glands are also present in the labial gland sample, subtracting genes expressed in thoracic feet was expected to facilitate identification of unique genes expressed solely in the labial glands.

### In situ hybridization

In situ hybridization of the candidate genes identified in the RNAseq data was done to confirm that they were expressed by the labial gland and not in surrounding tissues. Predicted sequences from the candidate genes were retrieved at ENSEMBLE Metazoa (https://metazoa.ensembl.org/Lepeophtheirus_salmonis/Info/Index), and gene specific primers were designed based on these sequences and BLASTED in the salmon louse genome to ensure specificity. Single stranded digoxigenin (DIG) labelled antisense and sense RNA probes of the selected genes were prepared by in vitro transcription using the DIG RNA Labelling Kit (Roche), with purified PCR products that included T7 promoters (TAATACGACTCACTATAGGGAGA) as templates (Primers listed in Table 1). PCR was performed as previously described using Q5^®^ High-Fidelity DNA Polymerase, and the products were purified with the GenElute ^(^™^)^ PCR Clean-Up Kit (Sigma-Aldrich).

**Table 1:** Sequences of primers used for characterization of LsLGPs.

Gene	Forward (5ʹ → 3ʹ)	Reverse (5ʹ → 3ʹ)	Use
LsLGP1L	AAACAATGGCTATAAAATGAATAATCCCATT	GAATAACCTCGTCATTCAGAATAACACCCT	RACE
LsLGP1	AAAACGAAGAGACAGTTGTCCCAAATT	CGTCATTCAGAATAACATCGTTTTTCTC	RACE
LsLGP1/1L	GGAGTATACAAGTACATCATGAAAGGAACA	CAGAAAGATATTGTGACATTTAGTTCAAAA	ORF
LsLGP1/1L	ATTCTTAATGAAGAATCTGGTGAGGGA	TTTTGAACTAAATGTCACAATATCTTTCTG	IS/RNAi
LsLGP1L	AAACAATGGCTATAAAATGAATAATCCCATT	GAATAACCTCGTCATTCAGAATAACACCCT	qPCR
LsLGP1	AAAACGAAGAGACAGTTGTCCCAAATT	CGTCATTCAGAATAACATCGTTTTTCTC	qPCR
LsLGP2	TGCACCTGCACCAGCACCTGCACCA	TGGTGCTGGTGGCGGTGCAATTCCA	RACE
LsLGP2	TGTTTTAAACTCAATGATGCATTGGCAACC	TTTAATGATCGAAAACGATGGGTAC	ORF
LsLGP2	GCAAATCCGGGAAAAGAAATCCAAG	TCGAAAACGATGGGTACGTCTC	IS/RNAi
LsLGP2	GCTCTCCTTAGTATCTTTATTTTGGGCTCC	ACACGTAAGCACAGCTTGTGGATGC	qPCR
LsLGP3	CCAGTGGGCTCCAATCCTAGTGAA	AGTTTGGGCTGTTGTTCCGGTT	RACE
LsLGP3	ATGAAACTAATATTTTCAAGTTTGATTACA	TTAGATATCTGTGTAATAAGTTTGGGCTGT	ORF
LsLGP3	CCAGTGGGCTCCAATCCTAGTGAA	AGTTTGGGCTGTTGTTCCGGTT	IS/RNAi
LsLGP3	TCAAGAAGGATTTGAAAAGTTGACGAATG	TGCCCGATTGTTCCATAATTCCGTT	qPCR
LsLGP3	CAGTCAGCTCTTCTAGTCAGCACGGTATTCGAGTGAAG		RecLGP4
LsLGP4	GAACAACGCAATCTGGACAA	CGGAGAGTACTTTAACATGAGTCA	RACE
LsLGP4	TGTTTTTTCAAGATCGACTCTTCCAATGG	TTAATCACTCTCGGAGAGTACTTTAACATG	ORF
LsLGP4	GAACAACGCAATCTGGACAA	CGGAGAGTACTTTAACATGAGTCA	IS/RNAi
LsLGP4	TGTTTTTTCAAGATCGACTCTTCCAATGG	AGCACCTTTCTTTTTTGCAAGTTCCATA	qPCR
LsEF1α	GGTCGACAGACGTACTGGTAAATCC	TGCGGCCTTGGTGGTGGTTC	qPCRref (ref)
LsADT3	CTGGAGAGGGAATTTGGCTAACGTG	GACCCTGGACACCGTCAGACTTCA	qPCRref (ref)

Primers were used in rapid amplification of cDNA ends (RACE), confirming the open reading frame (ORF), synthesis of RNA probes for in situ hybridization (IS) and double stranded RNA for RNA interference (RNAi), production of recombinant LsLGP3 (recLGP3), and for real time RT-PCR both target genes (qPCR) and reference genes (qPCRref). Gene name abbreviations: *Lepeophtheirus salmonis* (Ls), labial gland protein (LGP), elongation factor (EF), and adenine nucleotide translocator (ADT).

A pre-adult II female *L. salmonis* were fixed in phosphate-buffered 4% paraformaldehyde (pH 7.4) for 24 h at 4 °C. Subsequently, specimens were processed with the Histokinette 2000 (Reichert-Jung) where they were washed in PBS, dehydrated through a graded ethanol series, and embedded in paraffin wax. Horizontal sections, 4.0 μm thick, were cut with a Leica RM 225 microtome (Leica Microsystems). In situ hybridization was performed according to Dalvin et al.^64^, with some modifications as described earlier^65^. Additionally, the proteinase K treatment was prolonged to 18 min. Antisense and sense probe were applied to adjacent sections at each round of hybridization, to control for unspecific hybridization. Adjacent sections were also stained with hematoxylin (Shandon Instant Hematoxylin, Thermo Scientific) for 2.5 min and 1.5 min with 1% erythrosine (Certistain, Merck). HE stained sections were mounted in Histomount (Invitrogen).

### Cloning and sequencing

Once genes were confirmed to be expressed in the labial glands, primers for rapid amplification of cDNA ends (RACE) were designed from the predicted sequences (Table 1). RACE (5ʹ and 3ʹ) was performed for all genes using the SMARTer™ RACE cDNA amplification kit according to suppliers’ instructions (Clontech). RACE PCR products were cloned using the TOPO TA Cloning^®^ Kit for sequencing with One Shot™ TOP10 Chemically Competent *E. coli* (Thermo Fisher). Colonies were used as templates for PCR reaction using the Q5^®^ High-Fidelity DNA Polymerase kit according to suppliers’ recommendation (BioLabs), applying M13 forward and reverse primers (Cycles were: initial denaturation 98 °C for 30 s, 30 cycles of 98 °C for 10 s, 55 °C for 30 s and 72 °C for 30 s/kb, and a final extension for 2 min at 72 °C). PCR products were further purified by ExoSAP-it (Affymetrix) and sequenced using the BigDye Terminator v3.1 Cycle Sequencing kit from Applied Biosystems. Sequencing was completed on an ABI prism 7700 automated sequencing apparatus at the University of Bergen sequencing facility. The open reading frame (ORF) was further confirmed by one directly sequenced PCR as described above for colonies in 25 μl reactions, applying gene specific primers and 1 μl cDNA from copepodid or adult lice.

Sequences were assembled and translated using Vector NTI Advance 10 software (Invitrogen). Sequence similarity search of ORFs against GenBank was done using NCBI BLAST in tblastn (https://blast.ncbi.nlm.nih.gov/Blast.cgi). ORFs were aligned in Clustal Omega (https://www.ebi.ac.uk/Tools/msa/clustalo/). Location of domains was predicted by InterProScan (http://www.ebi.ac.uk/interpro/search/sequence/), and signal peptides in the SignalP-5.0 server (https://services.healthtech.dtu.dk/service.php?SignalP-5.0). ProtParam was used to compute theoretical pI and molecular weights (https://web.expasy.org/cgi-bin/protparam/protparam), and glycosylation sites were predicted in the NetNGlyc 1.0 server (https://services.healthtech.dtu.dk/service.php?NetNGlyc-1.0).

### Real time RT-PCR

Real time RT-PCR was performed with 1× PowerUp™ SYBR Green Master Mix (Thermo Fisher Scientific), 500 nM forward and reverse primers (Tables 1 or 2) and 2 µl diluted cDNA in 10 µl reactions. Samples were always run in duplicate (technical replicates) on the Applied Biosystems 7500 Real-Time PCR System under standard conditions (50 °C for 2 min, 95 °C for 2 min, 40 cycles of 95 °C for 15 s and 60 °C for 1 min, followed by a melt curve analysis at 60–95 °C). A five-point standard curve of fourfold dilutions was made for each assay to calculate PCR efficiencies, given by the equation E% = (10^1/slope^ − 1) × 100^66^. Salmon louse cDNA were further diluted 1:10, while cDNA from salmon skin or primary leucocytes were diluted 1:5. The relative differences in threshold cycle between the target gene and the geometric mean of the reference genes (ΔCT) and expression relative to a calibrator (ΔΔCT) were calculated, transformed by the equation 2^−ΔΔCT^^67^. The reference gene expression was evaluated in all datasets prior to calculation to ensure a stable expression (Supplementary Fig. S2).

**Table 2:** Sequences of primers used for real time RT-PCR analyzing immune responses in Atlantic salmon.

Gene	Forward (5ʹ → 3ʹ)	Reverse (5ʹ → 3ʹ)	Accession no
C3a	ATTCTTCCCCTCCACTCCCTCG	CGATTTGGTCGTCAAGCCAGG	XM_014186867
IL1β	GCTGGAGAGTGCTGTGGAAGA	TGCTTCCCTCCTGCTCGTAG	XM_014170479
IL4/13A	CGTACCGGCAGCATAAAAATCACCATTCC	CCTTGCATTTTGTGGTGGTCCCA	NM_001204895
IL6	ACCAACAGTTTGTGGAGGAGTTTCAGAAGC	CCTGCAGACATGCCTCCTTGTTG	KJ425513
IL8	GCATCAGAATGTCAGCCAGCC	ACGCCTCTCAGACTCATCCC	NM_001140710
IL10	ATGAGGCTAATGACGAGCTGGAGA	GGTGTAGAATGCCTTCGTCCAACA	XM_045705802
TNFα	CACTGCCACCAAGAGCCAAG	CGCCAGTTGTCATCGCATACC	DQ787157, DQ787158
IFNγ	ATGGATGTGTTATCAAGGGCTGTGATGTG	CAGCTGGTCCTTGGAGATCTTATAGTGGAC	AY795563, XM_045698695
MMP13	ACTCTTTGCCAATATCGCCACCCA	TGGGCCCTCGTTTGAACGCA	BT058668
MHCII	GGACGTGAGGTGAAGTCTGATGTGACC	CTGATGTGCTCCACCATGCAGGA	BT058598
TCRα	ATGAGCCATCCTACTACACGTTGAACTCAA	CACTCTGGTGGCCTCTGTATTGTTGAAGAC	BT057540
CD4	GAGTACACCTGCGCTGTGGAGT	GGTTGACCTCCTGACCTACAAAGG	EU585750
CD8α	TAGAGTGCAAGACAACGCTGGAATGGA	TCTCGAGCCTTTTTGAAAGCCTTCAG	AY693393, AY701521
NCCRP1	AATCCTGCGCCTCACGGTGTGAGTC	GCGAGGAGGTCCTTCTGGTGGAAAC	NM_001166257
IgD	CACCAGGAGGAAAGTTTGGCATCA	CCCCAAGGAGCTCTGGTTTGGA	AF141606
IgM	TGAGGAGAACTGTGGGCTACACT	TGTTAATGACCACTGAATGTGCAT	BT058539
IgT	GGTGGTCATGGACGTACTATTT	CCTGTGCAGGCTCATATCTT	GQ907004
EF1α	CACCACCGGCCATCTGATCTACAA	TCAGCAGCCTCCTTCTCGAACTTC	BT043567
TRIM16	TTACTGTAGGAGCTGTATTGAGGGCTGCTG	TTCTCCACCAGCTCAGCCAACATG	XM_014170167

Gene name abbreviations: complement component (C), interleukin (IL), tumor necrosis factor (TNF), interferon (IFN), matrix metalloproteinase (MMP), major histocompatibility complex (MHC), T-cell receptor (TCR), cluster of differentiation (CD), non-specific cytotoxic cell receptor (NCCR), immunoglobulin (Ig), elongation factor (EF), and tripartite motif-containing (TRIM) protein.

### RNA interference and infection studies

RNA interference (RNAi) followed by infestation studies were performed to analyse the functional role of the labial gland proteins in vivo. Double stranded RNA (dsRNA) was produced using MEGAscript^®^ RNAi Kit (Ambion) according to supplier’s instructions with primers for the LsLGPs listed in Table 1 and for the control fragment, CYP, see Dalvin et al.^68^. RNAi on salmon louse nauplii was performed by soaking as previously described by Eichner et al.^32^. Around 60–100 nauplius I larvae, all from the same egg string, were incubated overnight (17 h) in 150 µl of seawater containing 20 ng/µ1 control or labial gland gene dsRNA (LsLGP1–3). Thereafter, all animals within a treatment group were pooled and kept in flow through incubators until at least one day after the copepodid stage was reached. For pre-adult (LsLGP5) and adult lice (LsLGP4 and 5), 50 μl of LsLGP or control dsRNA solution was stained with 1 μl Bromphenol blue to enable us to see that approximately 600 ng dsRNA were injected dorsally into the haemocoel of the cephalothorax. Pre-adult lice were incubated off-host for 4 h prior to infestation, while adult lice were incubated 4 days off-host to allow knock down (KD) of target mRNAs prior to infestation as the sampling and immune analysis were to be conducted 3 days post infestation.

For immune studies, Atlantic salmon (average weight around 150 g) were kept individually in single tanks at 12 °C^69^. In each infestation experiment, three groups of fish were included (N = 6–8): an untreated group of fish, fish infested with control lice and fish infested with KD animals. Around 80 copepodids/fish or 10 adult females were added, and scaled skin samples were taken at and away from the attachment site of the lice 3 days post infestation (dpi). Immune responses were measured analyzing the transcript level of Atlantic salmon immune genes listed in Table 2. The lice number was recorded, and KD in lice transcript level was also measured and confirmed to be below 90% before subsequent analysis.

Injection of pre-adult lice II were conducted to analyse the effect of LsLGP4 KD on coagulation, feeding and development. The pre-adult lice (10/fish) were held on fish (N = 3) for 32 days, to allow for the second egg string to be extruded. After 32 days, the lice were counted and carefully removed from the fish to analyse KD of LsLGP4 transcript level. Pictures was taken to allow for size measurements in image J and visual inspection of gut blood content. All KD experiments were repeated twice with different batches of fish.

### Heterologous expression and synthesis of labial gland proteins

The open reading frame (ORF) of *LsLGP3* was used as templates for gene synthesis, and the gene was codon optimized to maximize expression in *E. coli* (GenScript). The gene was flanked by *Sap*I restriction sites and delivered in a customized SapI-free plasmid with kanamycin selection marker (GenScript). Gene fragments excluding the signal peptides were sub-cloned into expression vectors, p1 and p12, as previously described by Bjerga et al.^70^, introducing an N-terminal or a C-terminal hexahistidin-tag, respectively. Before heterologous expression in *E. coli* MC1061, the inserts were sequenced to verify the ORF. Protein expression was induced after 2.5 h at 37 °C with 0.1% l-arabinose (Sigma) and produced over-night at 20 °C. Recombinant soluble LsLGP3 (recLGP3) was purified with the Ni–NTA Fast Start Kit (Qiagen). The buffer was changed to 1× PBS with a Pierce™ Protein concentrator, 10K MWCO (Thermo Scientific). The purity of recLGP3 was analyzed by Coomassie staining of SDS-PAGE gels (Supplementary Fig. S3), and the concentration measured on a Nanodrop Spectrophotometer (absorbance at 280 nm). Purified recLGP3 was stored at − 80 °C, and any precipitation removed by centrifugation (13,000×*g*, 10 min at 4 °C) before use. Moreover, the concentration of LPS-contamination was analyzed with the ToxinSensor™ Chromogenic LAL Endotoxin Assay Kit (GenScript^®^) according to the suppliers protocol. For LsLGP4, a synthetic LsLGP4 (synLGP4) was purchased (LeifTein). The purity of synLGP4 was analyzed with high pressure liquid chromatography to be 99.31%, and the peptide sequence was confirmed by Mass Spectrometry by the supplier.

### In vitro stimulation of primary head kidney leucocytes

Atlantic salmon primary head kidney leucocytes were stimulated with recLGP3 and synLGP4 to analyze their involvement in immune modulation. Each experiment was repeated twice for each protein to be tested, as follows.

The average fish weight was 520 g for recLGP3 experiments and around 600 g for synLGP4 experiments. The leucocytes were isolated from three fish in each experiment, on a discontinuous Percoll gradient (Fisher) as previously described^71^, using L-15 medium (Gibco) supplemented with 10% Fetal bovine serum (Merck), heparin (Leo Pharma) and 1% penicillin (Merck). The leucocytes were counted manually with a hemocytometer, and cell viability evaluated using 0.04% Trypan Blue Stain (Gibco). Cells from each fish were plated into 96-well culture plates (Falcon), with a density of approximately 1 × 10^6^ cells/well in 100 μl supplemented L15-medium. For recLGP3-stimulation, eight wells per fish where plated, given 1 × PBS (control), 1 μM/well LPS (Merck) or three different concentrations of protein (150, 112.5 and 75 μg protein/well). RecLGP3 was diluted to different concentrations in PBS to a final volume of 100 μl and added to the cells, whereas 100 μl PBS with or without LPS was used for control cells. For synLGP4-stimulation, four wells/fish was plated given control and the three different concentrations of the proteins as for recLGP3. SynLGP4 was diluted to different concentrations in ddH_2_O to a final volume of 30 μl and supplemented with L-15 medium to a final volume of 100 μl. The control cells received 30 μl ddH_2_O in 70 μl L-15 medium.

The cells were incubated at 16 °C. After four and 20 h, the L15-medium was removed and centrifuged for 2 min at 460×*g*, 4 °C, to harvest the non-adherent cells. The supernatant was removed, and the pellet was dissolved in 300 µl TRI reagent (Sigma). TRI reagent, 200 µl, was added to the adherent cells immediately after removal of the L15-medium and transferred to the non-adherent cells after repetitive mixing and scraping with the pipette. The cells were frozen in Tri reagent at − 80 °C until total RNA isolation as described in “Source of *L. salmonis*” section, after addition of 500 μl TRI reagent to the homogenized cells. Immune responses were measured analyzing the transcript level of Atlantic salmon immune genes listed in Table 2.

### Blood coagulation measurement

To determine the anti-coagulation ability of recLGP3 and synLGP4, prothrombin time (PT) was determined by a modified Quick one-stage method^72,73^. Blood was collected from three Atlantic salmon (between 900 and 1400 g) into a syringe pre-filled with 10 mM sodium citrate (Merck) to a final ratio of one volume sodium citrate to nine volumes blood. The blood was centrifuged for 15 min at 180×*g*, 4 °C to collect the plasma. For each fish, 100 µl plasma was mixed with 100 µl 25 mM CaCl_2_ (Merch) and 100 µl rabbit Thromboplastin (Sigma Aldrich, 100 µg/µl in 10 mM CaCl_2_), and aliquoted into eight tubes at room temperature. Either 50 or 75 µg recLGP3 or 75 µg synLGP4 dissolved in 15 µl ddH_2_O was added to the plasma mixture, or 15 µl ddH_2_O for control plasma, all in duplicates. This setup gave two technical replicates per fish per treatment. The time until clot formation was measured while the tubes were continuously tilted back and forth 90 degrees but stopped after 45 min if no clotting was visible (only synLGP4 treated group). This experiment was repeated three times for LsLGP4 and twice for LsLGP3.

### Statistical analysis

All statistical analysis were performed in GraphPad prism 8.0.1 (GraphPad Software). A student’s t-tests were used to test for differences when only two groups are compared (control versus treatment, α = 0.05). A one-way ANOVA was used to test for differences when more than two groups are compared (α = 0.05), using a Tukey’s multiple comparisons post hoc test.

## Supplementary Information

Supplementary Legends.

Supplementary Figure S1.

Supplementary Figure S2.

Supplementary Figure S3.

## Data Availability

The datasets generated and/or analyzed during the current study are available from the corresponding author on reasonable request.
