# Supplementary material for: Small, charged proteins in salmon louse (Lepeophtheirus salmonis) secretions modulate Atlantic salmon (Salmo salar) immune responses and coagulation
Source: Sci Rep. 2022 May 14;12:7995. doi: 10.1038/s41598-022-11773-w (PMC9107468; doi:10.1038/s41598-022-11773-w)
Supplement: Supplementary file 1 — Supplementary Legends. [file 41598_2022_11773_MOESM1_ESM.pdf]

Small, charged proteins in salmon louse (*Lepeophtheirus salmonis*) secretions modulate Atlantic salmon (*Salmo salar*) immune responses and coagulation

Aina-Cathrine Øvergård[1*], Helena M.D. Midtbø[1], Lars A. Hamre[1], Michael Dondrup[2], Gro E. K. Bjerga[3], Øivind Larsen[3], Jiwan Kumar Chettri[4], Kurt Buchmann[4], Frank Nilsen[1] and Sindre Grotmol[1].

Supplementary figure legends

Supplementary figure S1

Relative transcript level ($2^{-\Delta\Delta Ct}$ ± SD) of selected Atlantic salmon immune gene transcripts in head kidney leucocytes after a 4-hour stimulation with synthetic LsLGP4 (N = 3). Three quantities of protein were applied, 150, 112.5 and 75 µg/well. The immune gene expression were related to *elongation factor 1 alpha* (*elf1α*) and tripartite motif-containing protein (TRIM) (ΔCt), using the expression in control cells as calibrator (ΔΔCt). A significant difference ($p \geq 0.05$) between control and synLGP4 treated leucocytes were not found.

Supplementary figure S2

To evaluate the stability of the reference genes, the threshold cycler (CT) values for the two reference genes, elongation factor 2 alpha (EF1α) and tripartite motif-containing protein 16 (TRIM16) and the geometric mean of the two was plotted for each sample used in the knock-down study. The mean for each gene is indicated with a stippled line (EF1α 16.9±0, GeoMean 18.2±0.25 and TRIM16 19.66±0.42).

Supplementary figure S3

Coomassie staining of SDS-PAGE gel after His-tag purification of recombinant LsLGP3 (recLGP3). Picture was taken in a GelLogic 212 PRO. Lane M – marker, FT – flow trough, E1-5 – eluate 1-5. Expected size of recLGP3 is 20.95 kDa.
