# Supplementary figures and images for: Small, charged proteins in salmon louse (Lepeophtheirus salmonis) secretions modulate Atlantic salmon (Salmo salar) immune responses and coagulation
Source: Sci Rep. 2022 May 14;12:7995. doi: 10.1038/s41598-022-11773-w (PMC9107468; doi:10.1038/s41598-022-11773-w)

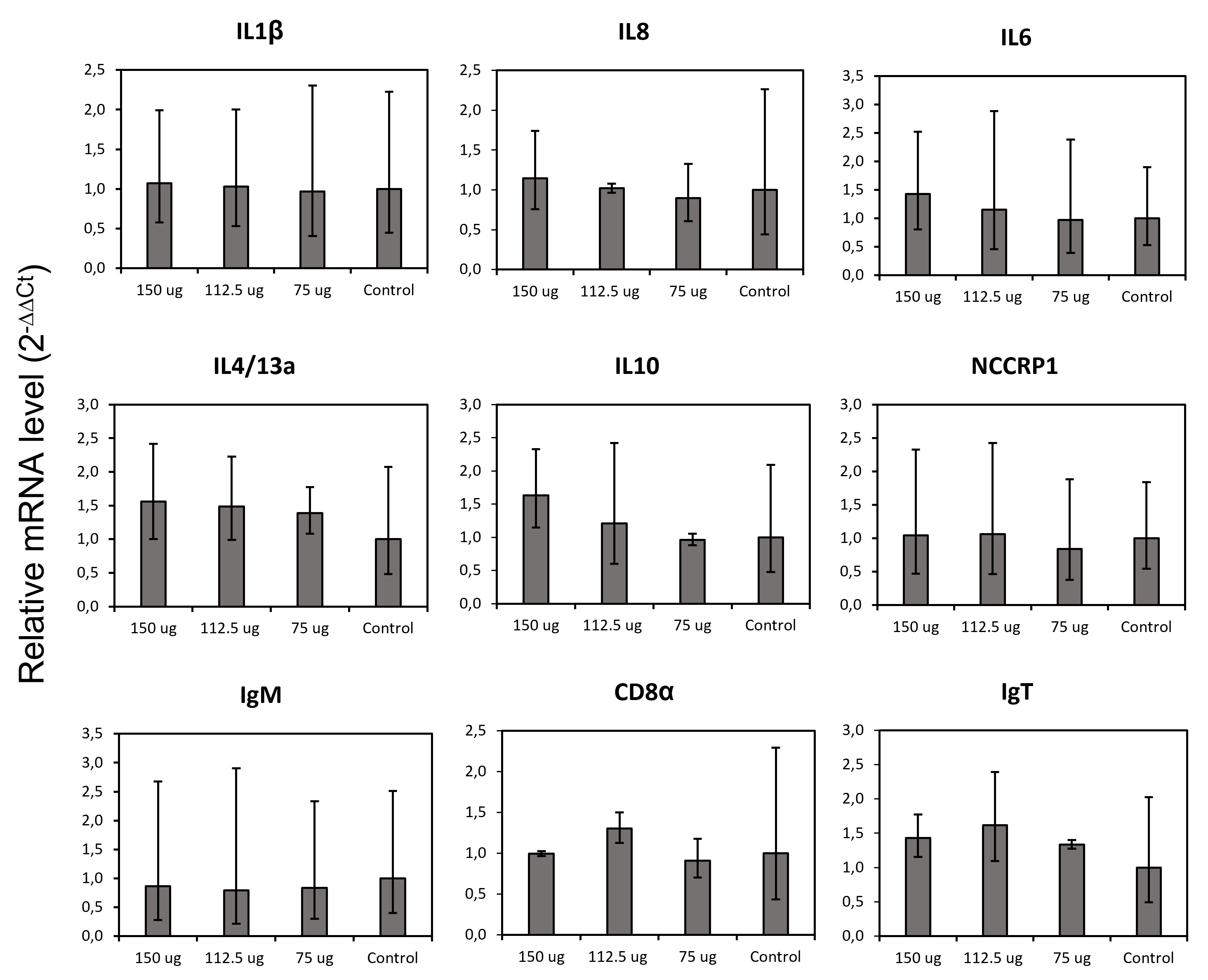

Supplement: Supplementary file 2 — Supplementary Figure S1. [file 41598_2022_11773_MOESM2_ESM.tif]

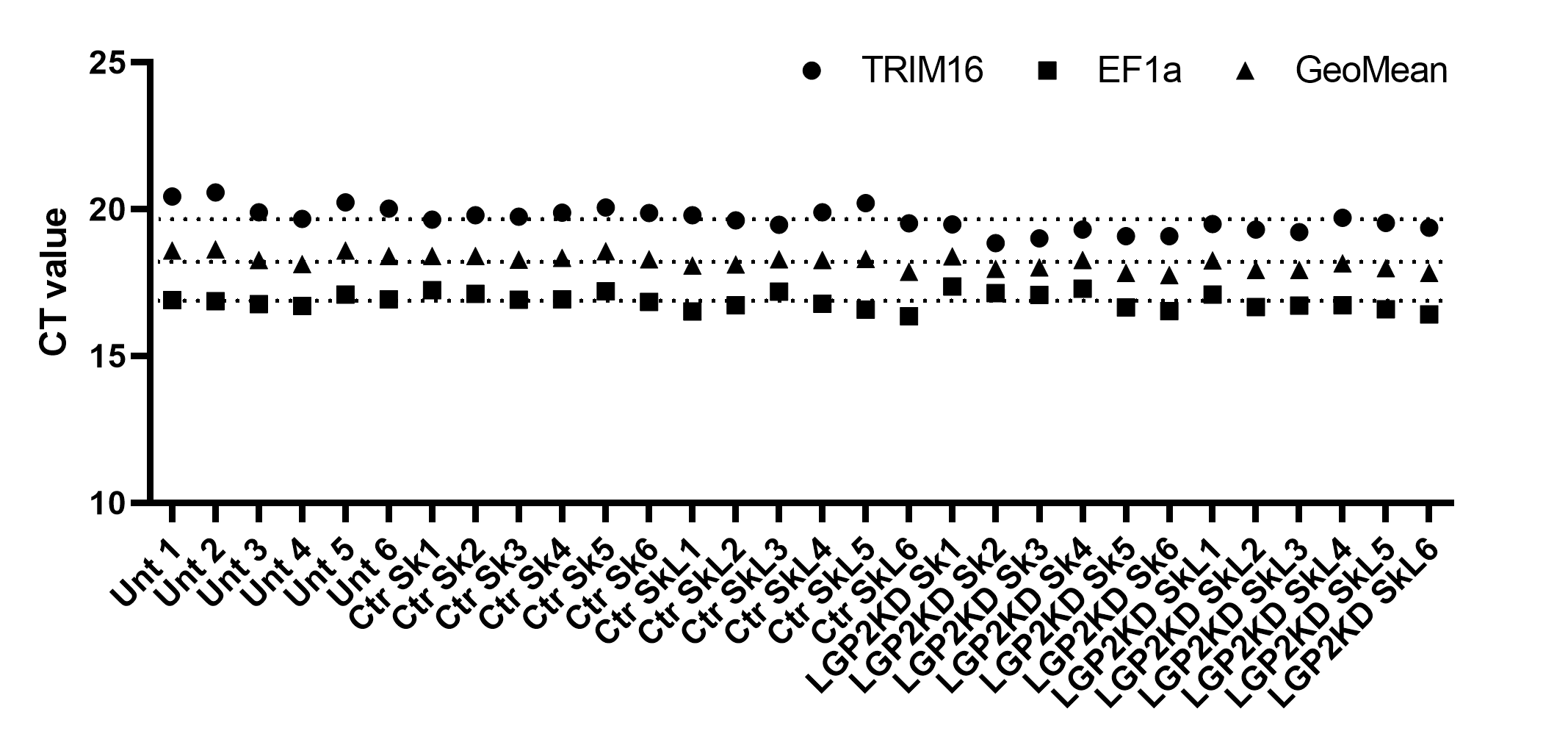

Supplement: Supplementary file 3 — Supplementary Figure S2. [file 41598_2022_11773_MOESM3_ESM.tif]

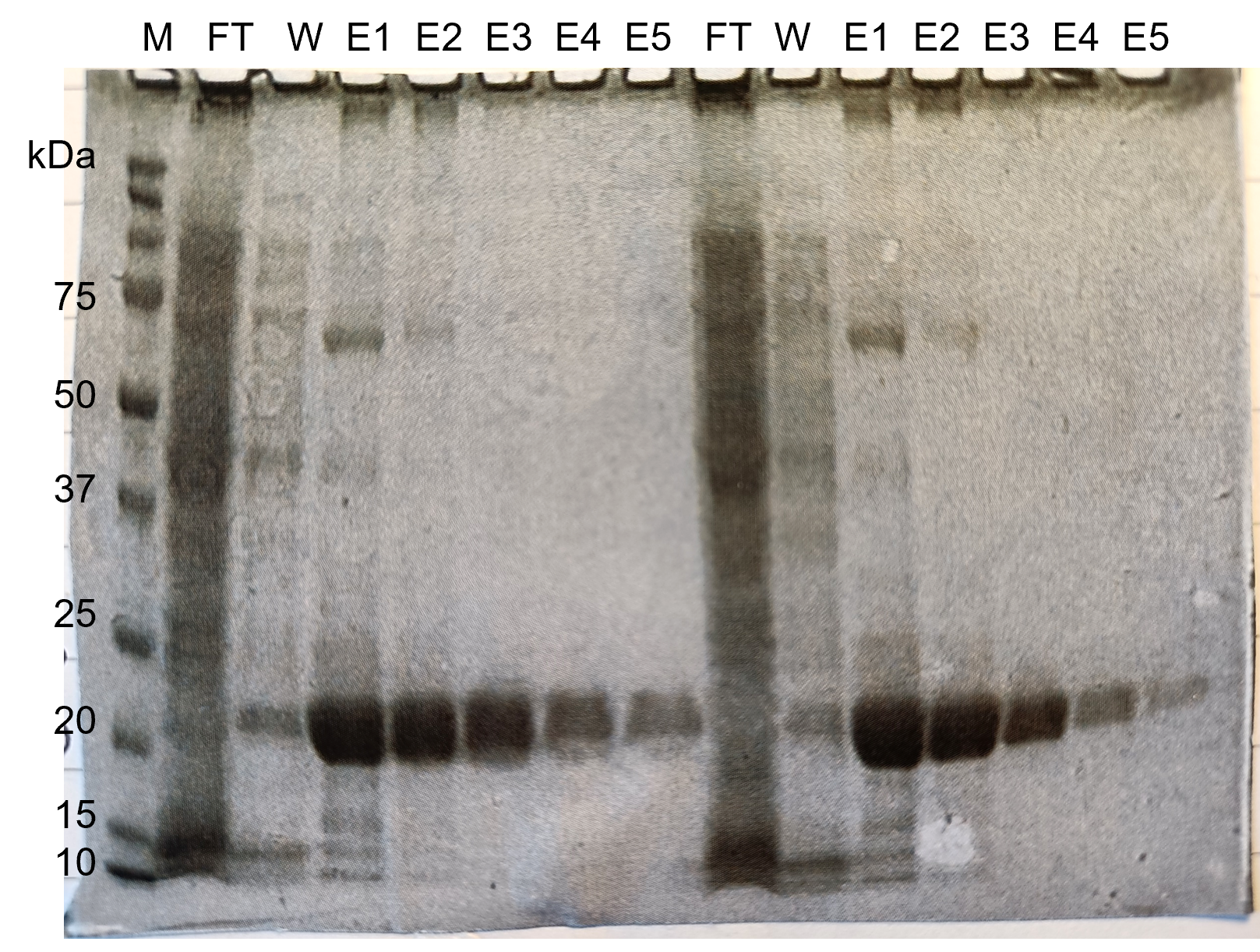

Supplement: Supplementary file 4 — Supplementary Figure S3. [file 41598_2022_11773_MOESM4_ESM.tif]
